# Phages released from *Acidithiobacillus ferrooxidans* enhance chalcopyrite bioleaching by alleviating passivation and promoting sulfur turnover

**DOI:** 10.1007/s44307-026-00122-x

**Published:** 2026-07-27

**Authors:** Zhaoyue Yang, Zhenghua Liu, Delong Meng, Zhendong Yang, Kuojun Hu, Zhuzhong Yin, Ling Xia, Ibrahim Ahmed Ibrahim, Xiangdong Xiao, Xueduan Liu, Huaqun Yin

**Affiliations:** 1 School of Minerals Processing and Bioengineering, Central South University, Changsha, 410083 China; 2 School of Architecture and Civil Engineering, Chengdu University, Chengdu, 610106 China; 3 Hubei Key Laboratory of Mineral Resources Processing and Environment, School of Resources and Environmental Engineering, Wuhan University of Technology, Wuhan, 430070 China; 4 Central Metallurgical Research and Development Institute, Cairo, 11421 Egypt; 5 Hunan Yama Biotechnology Co., Ltd., Changsha, 410000 China

**Keywords:** Bioleaching, Bacteriophages, Chalcopyrite passivation, Microbial communities, Sulfur turnover

## Abstract

Chalcopyrite is the most abundant copper-bearing sulfide mineral, characterized by a stable crystal structure. Bioleaching of chalcopyrite is inherently slow and further constrained by surface passivation layers during prolonged bioleaching. In this study, bacteriophages released from *Acidithiobacillus ferrooxidans* were introduced to promote chalcopyrite bioleaching. Mineralogical characterization, microbial profiling, and functional gene analysis were employed to reveal the underlying mechanisms. One day after phage introduction, phage abundance was 9.42 × 10^5^ VLPs mL^−1^ in the phage-treated group, 150-fold higher than in the control (6.26 × 10^3^ VLPs mL^−1^). Over a 60-day leaching period, the copper leaching efficiency of the phage-treated group was 31.72%, significantly higher than that of the control (20.43%). Phage introduction alleviated surface passivation, suppressed elemental sulfur (S^0^) and jarosite-type deposition, and increased the apparent product-layer diffusion rate constant 3.60-fold. Bacterial diversity and the relative abundance of heterotrophic bacteria were elevated. The increase in heterotrophic bacteria may alleviate the inhibitory effects of organic matter on autotrophic bioleaching microorganisms. Genes associated with the Sox system, dissimilatory sulfur metabolism, and organic sulfur transformation were significantly enriched in the phage-treated group, consistent with enhanced S^0^ turnover and reduced surface accumulation. Together, these results indicate that phage introduction enhances chalcopyrite bioleaching by alleviating surface passivation and promoting sulfur turnover through coupled interfacial and microbial regulation. This study provides a novel approach for sustainable intensification of bioleaching of refractory sulfide minerals.

**Supplementary Information:**

The online version contains supplementary material available at 10.1007/s44307-026-00122-x.

## Introduction

With high-grade copper reserves rapidly depleting, copper supply increasingly relies on the processing of refractory resources, especially chalcopyrite (CuFeS_2_), which hosts approximately 70% of global copper resources (Velásquez-Yévenes et al. 2022). Bioleaching is a key technology for the sustainable extraction of low-grade chalcopyrite. However, bioleaching of chalcopyrite suffers from a low leaching rate under mesophilic conditions, commonly below 30% even after prolonged operation (Abdollahi et al. 2014). This is because chalcopyrite possesses a highly stable Cu-Fe-S crystal lattice that limits bacterial attack. Once the surface lattice is disturbed by microbial attachment and ferric oxidation, high-energy defective sites may undergo relaxation or reconstruction. Meanwhile, the sluggish and incomplete oxidation of sulfide species favors the accumulation of elemental sulfur, polysulfides, and jarosite precipitates on the mineral surface or within the extracellular polymeric substance (EPS) layer. These passivation layers greatly impede mass transport and interfacial electron transfer across the biofilm-mineral interface, thereby suppressing mineral dissolution kinetics (Vera et al. 2022; Zhao et al. 2019). Therefore, overcoming interfacial passivation has become a central scientific and technological challenge in chalcopyrite bioleaching.

To mitigate passivation and enhance chalcopyrite bioleaching, various physicochemical strategies have been explored. For instance, regulation of solution pH (Liu et al. 2016) and redox potential (Sandström et al. 2005) can inhibit secondary precipitate formation, while catalytic agents such as Ag^+^ (Nourmohamadi et al. 2022; Zhao et al. 2022), activated carbon (Oyama et al. 2021), and organic electron mediators (Liu et al. 2024, 2019; Yang et al. 2017) facilitate electrochemical reactions and reduce passivation layer accumulation. While effective, these approaches are constrained by economic feasibility and process scalability in industrial applications. Ecological regulation of microbial communities offers a complementary strategy (Yan et al. 2025). For example, progressively increasing the proportion of sulfur-oxidizing bacteria (i.e., *Acidithiobacillus thiooxidans*) relative to iron-oxidizing bacteria (i.e., *A. ferrooxidans*) enhanced sulfur oxidation and reduced passivation (Feng et al. 2015). However, such approaches rely on exogenous microbial inocula, which often underperform during long-term operation due to functional decline.

Recent studies have reported abundant and diverse bacteriophage (phage) populations in bioleaching systems (Gao et al. 2020, 2022; Han et al. 2024; Karmakar et al. 2024). Our previous study demonstrated that phages regulated microbial communities together with environmental factors in copper mine bioleaching solutions (Liu et al. 2023, 2025). Phages can disrupt biofilm architecture through selective infection of host cells (Chang et al. 2022; Visnapuu et al. 2022) and enhance sulfur oxidation in host cells through auxiliary metabolic genes (Bi et al. 2025; Kieft et al. 2021), suggesting that phage activity could improve bio-oxidation of sulfide ores. Based on these findings, we propose a phage-mediated strategy to alleviate interfacial passivation and intensify chalcopyrite bioleaching.

In this study, we investigated the effects of phages released from *Acidithiobacillus ferrooxidans* on chalcopyrite bioleaching performance and explored the underlying mechanisms. We aimed to (i) determine whether phages can regulate mineral-microbe interfacial reactions and enhance copper leaching efficiency, and (ii) elucidate how phages alter microbial community structure and sulfur-related functional potential. Our findings demonstrate that phage-mediated regulation effectively enhances chalcopyrite bioleaching efficiency, providing a novel approach for sustainable intensification of bioleaching.

## Materials and methods

### Chalcopyrite samples

Chalcopyrite samples were obtained from the Dabaoshan mine (Guangdong, China) and ground to a particle size fraction of 38–75 µm. The elemental composition was analyzed using inductively coupled plasma optical emission spectrometry (ICP-OES; SPECTRO BLUE SOP, Germany) and an infrared carbon–sulfur analyzer (LECO CS744, USA). The samples were mainly composed of Cu (32.7 wt.%), Fe (30.7 wt.%), and S (34.4 wt.%), with minor Ca (0.11 wt.%) and trace elements.

### Microbial consortium enrichment

The microbial consortium was enriched from acid mine drainage (AMD) collected from the same site, with an initial pH of 2.06. The AMD sample was inoculated at 10% v/v into basal salts solution (BSS; pH 2.0, adjusted with 3 M H_2_SO_4_). The BSS contained the following components in g L^−1^: (NH_4_)_2_SO_4_, 3.0; MgSO_4_∙7H_2_O, 0.5; Na_2_SO_4_∙10H_2_O, 0.15; KH_2_PO_4_, 0.05; KCl, 0.1; and Ca(NO_3_)_2_∙4H_2_O, 0.014. To maximize microbial diversity, three enrichment cultures were established using different energy substrates: (i) Fe^2+^ only, supplemented with 44.7 g L^−1^ FeSO_4_∙7H_2_O; (ii) S^0^ only, supplemented with 10 g L^−1^ elemental sulfur; and (iii) mixed substrates, supplemented with 22.4 g L^−1^ FeSO_4_∙7H_2_O, 5 g L^−1^ S^0^, and 0.2 g L^−1^ yeast extract. Cultures were incubated at 30 °C with rotary shaking at 180 rpm. After three sequential subcultures at 48 h intervals, the three enrichment cultures were adjusted to the same cell density and pooled at equal volumes to generate a composite AMD-derived consortium with an initial cell density of approximately 1 × 10^8^ cells mL^−1^. 16S rRNA gene sequencing showed that *Acidithiobacillus* was the dominant genus in the inoculum (48.5%), followed by *Acidiphilium* (4.4%).

### Phage induction, purification, and quantification

Phages were obtained from *Acidithiobacillus ferrooxidans* through mitomycin C (MMC)-mediated prophage induction. The host strain was cultured in 150 mL of Fe^2+^-supplemented BSS at 30 °C and 180 rpm. When the culture reached the exponential phase, with a cell density of approximately 1 × 10^8^ cells mL^−1^, MMC (Aladdin, China) was added to a final concentration of 1.0 µg mL^−1^. Cell density was monitored by hemocytometer counting under a light microscope. A marked decrease in *A. ferrooxidans* cell density was observed after 4 h of induction, indicating successful prophage induction and lytic activity. Cell debris was then removed by filtration through a 0.22 µm membrane.

Phage particles in the filtrate were precipitated using polyethylene glycol 8000 (PEG 8000; 10% w/v) and NaCl (1 M) overnight at 4 °C, followed by centrifugation at 9000 rpm for 30 min at 4 °C. The pellet was resuspended in SM buffer. To remove free nucleic acids, DNase I was added to a final concentration of 10 µg mL^−1^ and incubated at 37 °C with shaking for 30 min. An equal volume of chloroform was then added to remove lipids and membrane debris, vortexed for 30 s, and centrifuged at 3000 rpm for 15 min. The upper aqueous phase containing phages was collected. The phage suspension was further purified by cesium chloride (CsCl) density gradient ultracentrifugation using a 1.35–1.70 g mL^−1^ CsCl gradient and a Beckman SW41Ti rotor at 180000 × g for 1 h at 4 °C. Visible opalescent bands containing phage particles were collected and dialyzed against SM buffer. Purified phage particles were visualized by transmission electron microscopy (TEM; Talos L120C, Thermo Fisher Scientific, USA) at an accelerating voltage of 80 kV.

Phage abundance was quantified as virus-like particles (VLPs) by SYBR Gold staining and epifluorescence microscopy. Briefly, phage suspensions were filtered onto 0.02 µm Anodisc membranes to retain viral particles. The membranes were stained with 2× SYBR Gold (Invitrogen, Thermo Fisher Scientific, USA) for 15 min and rinsed three times with PBS buffer to remove unbound dye. The stained membranes were transferred onto glass slides, mounted with antifade reagent, and observed using a Leica DM2500 fluorescence microscope at an excitation wavelength of 488 nm. Five randomly selected fields were counted per membrane, and phage abundance was calculated as VLPs mL^−1^.

### Bioleaching experiment

Bioleaching experiments were conducted in 250 mL Erlenmeyer flasks containing 150 mL of BSS and chalcopyrite at a pulp density of 1% w/v. The initial pH was adjusted to 2.0. This pulp density was selected to maintain a uniform mineral suspension, support microbial activity, and allow clear observation of passivation-layer development during bioleaching. Two treatments were established: (i) a control group (CK), inoculated with 10% v/v AMD-derived bacterial consortium without phage addition; and (ii) a phage-treated group (Af_phage), inoculated with the same consortium and amended with phages on day 24. Day 24 was selected as the onset of surface passivation, marked by visible precipitate accumulation on mineral surfaces (Fig. S1). This time point was chosen because the passivation layer was still loose and accessible to phages at this early stage.

Because conventional plaque assays were difficult to perform in the strongly acidic medium required for *A. ferrooxidans* cultivation, phage dosage was standardized using host biomass. Specifically, phage preparations were obtained from 150 mL of MMC-induced *A. ferrooxidans* culture (host cell density ~1 × 10^8^ cells mL^−1^ before induction), concentrated by PEG precipitation, treated with DNase I and chloroform, and resuspended in 1 mL of SM buffer (Section 2.3, before CsCl purification). This 1 mL suspension was added to each Af_phage flask. Because the 1 mL phage suspension was added to 150 mL of leaching medium, residual SM buffer salts, trace PEG, and DNase were diluted by approximately 150-fold, making a major abiotic contribution to the observed biological and interfacial effects unlikely. All flasks were incubated at 30 °C with rotary shaking at 180 rpm. All treatments were performed in three biological replicates (n = 3). Samples were collected periodically for physicochemical, mineralogical, and microbiological analyses. One day after phage introduction, phage abundance was determined by SYBR Gold staining and epifluorescence microscopy.

### Physicochemical and mineralogical characterization

The pH of the leachate was measured using a calibrated pH meter. Dissolved copper (Cu), iron (Fe), and sulfur (S) concentrations were quantified using ICP-OES (PerkinElmer Optima 5300DV, USA). Ferrous iron (Fe^2+^) was determined by the 1,10-phenanthroline spectrophotometric method, and sulfate (SO_4_^2−^) was measured using the barium chromate method. Ferric iron (Fe^3+^) was calculated as the difference between the total dissolved Fe and Fe^2+^, and the Fe^3+^/Fe^2+^ ratio was derived accordingly.

The copper extraction efficiency (*α*) was calculated according to Eq. (1):

1 $$\alpha =\frac{{C}_{\mathrm{t}}\times V}{m\times {w}_{\mathrm{C}\mathrm{u}}}\times 100\%$$

where *C*_t_ is the dissolved Cu concentration in the leachate at sampling time t (g L^−1^), *V* is the volume of the leachate (L), m is the initial mass of chalcopyrite added (g), and *w*_Cu_ is the Cu mass fraction of the chalcopyrite (32.7%, w/w).

To characterize the microbe-mineral interface, mineral residues were collected by centrifugation at 5000 rpm for 5 min, fixed with 2.5% glutaraldehyde, dehydrated through a graded ethanol series (30%–100%), and critical-point dried. Surface morphology was examined by scanning electron microscopy (SEM; TESCAN MIRA3, Czech Republic). Surface chemical states were analyzed using X-ray photoelectron spectroscopy (XPS; Thermo Fisher NEXSA, USA), and functional groups were characterized by Fourier transform infrared spectroscopy (FTIR; Nicolet iS50, Thermo Fisher Scientific, USA).

### DNA extraction and multi-omics sequencing

Microbial biomass was collected separately from the planktonic (P) and mineral-associated (M) phases. Planktonic cells were harvested by filtering leachates through 0.22 µm membranes. Mineral-associated cells were detached by vortexing mineral residues in sterile BSS (pH 2.0) and then collected by filtration through 0.22 µm membranes. The corresponding 0.22 µm filtrates from both phases were combined for phage particle concentration using the iron chloride (FeCl_3_) flocculation method, and viral flocs were collected on 0.8 µm membranes (John et al. 2011).

Genomic DNA from the 0.22 µm membranes was extracted using the E.Z.N.A. Soil DNA Kit (Omega Bio-tek, USA). The V3–V4 hypervariable region of the bacterial 16S rRNA gene was amplified using primers 341F and 806R (Zhang et al. 2024). PCR products were purified using the AxyPrep DNA Gel Extraction Kit (Axygen, USA) and sequenced on an Illumina MiSeq PE300 platform (Shanghai Biozeron, China). Raw reads were processed in QIIME2 using DADA2 for denoising and amplicon sequence variant (ASV) inference (An et al. 2025), and taxonomic assignment was performed against the SILVA 138 database (Bolyen et al. 2019; Callahan et al. 2016; Quast et al. 2013).

For metagenomic sequencing, the same DNA extracts were used to construct paired-end libraries using the NEXTFLEX Rapid DNA-Seq Kit (Bioo Scientific, USA) followed by sequencing on an Illumina NovaSeq 6000 platform (paired-end 150 bp). Reads were quality-filtered using fastp (v0.23.2) and assembled using MEGAHIT (v1.2.9) (Li et al. 2015).

Phage particles retained on 0.8 µm membranes were resuspended and further purified by sucrose cushion ultracentrifugation (28% w/w; 160000 × g, 2 h). Purified virions were treated with DNase I and RNase A prior to viral DNA extraction using the QIAamp MinElute Virus Spin Kit (Qiagen, Germany). Viral DNA was amplified by multiple displacement amplification using the Illustra GenomiPhi V3 Kit and sequenced on an Illumina PE150 platform. The purified phage preparation (Section 2.3) was also subjected to viral DNA extraction, amplification, and sequencing using the same procedure to characterize the introduced inoculum.

### Microbial community analysis and functional annotation

Viral contigs were identified using VirSorter2 (v2.2.3; default parameters) (Guo et al. 2021), and genome quality was assessed by CheckV (v0.8.1) to remove potential host contamination (Nayfach et al. 2021). Viral operational taxonomic units (vOTUs) were defined by clustering viral contigs using CD-HIT-EST (v4.8.1; parameters: -c 0.95 -aS 0.80) (Fu et al. 2012). Viral clusters were further classified using vConTACT2 (v0.11.3) based on gene-sharing networks (Jang et al. 2019).

Open reading frames (ORFs) of vOTUs were predicted using Prokka (v1.14.6), and basic genomic features, including GC content and coding density, were calculated for each vOTU (Seemann 2014). Functional annotation and KEGG Orthology (KO) assignment were conducted using EggNOG-mapper (v2.1.11; E-value ≤ 10^−3^ and best-hit) (Cantalapiedra et al. 2021; Huerta-Cepas et al. 2019). Sulfur and iron metabolism genes were annotated using SCycDB (Yu et al. 2021) and FeGenie (Garber et al. 2020), respectively. Antiphage defense systems were identified using DefenseFinder (v2.0.1) (Tesson et al. 2022). Gene abundances were calculated as reads per kilobase per million mapped reads (RPKM) using CoverM (v0.6.0) (Aroney et al. 2025).

### Statistical and ecological analysis

All statistical analyses were conducted in R (v4.5.2). Alpha-diversity indices, including observed richness and Shannon index, were calculated using the vegan package (v2.7–2) (Oksanen et al. 2025). Ecological assembly processes were quantified using the iCAMP package (v1.5.12) (Ning et al. 2020).

Co-occurrence networks were inferred separately for each treatment from matched bacterial ASV (16S rRNA) and viral OTU (vOTU) abundance profiles collected from three biological replicate flasks at two time points, yielding six longitudinal observations per treatment. Spearman correlations were calculated across these six matched observations and retained at |*r*|> 0.5 and *P* < 0.05. Network topological properties, including modularity and degree (Barberán et al. 2012), were calculated using the igraph package (Csárdi And Nepusz 2006). Niche differentiation between planktonic and mineral-associated taxa was assessed using a niche specificity index (Eq. 2):

2 $$Index= \frac{{A}_{\mathrm{p}\mathrm{l}\mathrm{a}\mathrm{n}\mathrm{k}\mathrm{t}\mathrm{o}\mathrm{n}\mathrm{i}\mathrm{c}}-{A}_{\mathrm{M}\mathrm{i}\mathrm{n}\mathrm{e}\mathrm{r}\mathrm{a}\mathrm{l}}}{{A}_{\mathrm{p}\mathrm{l}\mathrm{a}\mathrm{n}\mathrm{k}\mathrm{t}\mathrm{o}\mathrm{n}\mathrm{i}\mathrm{c}}+{A}_{\mathrm{M}\mathrm{i}\mathrm{n}\mathrm{e}\mathrm{r}\mathrm{a}\mathrm{l}}}$$

where *A*_planktonic_ and *A*_Mineral_ represent the mean relative abundance of each taxon in the planktonic and mineral-associated phases, respectively. Taxa were classified as planktonic specialists (*Index* > 0.5), mineral-associated specialists (*Index* < − 0.5), or generalists (|*Index*|≤ 0.5), and these classifications were visualized within the network. For time-point-specific comparisons, statistical significance between treatments was determined using Student’s *t*-test based on three biological replicate flasks (n = 3 per treatment). *P* values were Bonferroni-adjusted where indicated. All visualizations were generated using ggplot2 (v4.0.1) (Wickham 2016).

## Results

### Phage morphology, abundance, and genomic features

**Fig. 1:**
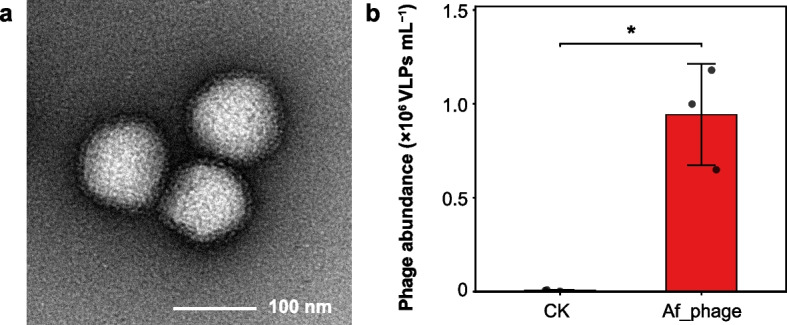
Morphological characterization and abundance of phages released from *Acidithiobacillus ferrooxidans*. **a** Transmission electron microscopy (TEM) image of purified phage particles showing icosahedral capsids. **b** Abundance of virus-like particles (VLPs) in the CK and Af_phage groups one day after phage introduction. Bars represent mean ± SD (n = 3). An asterisk indicates *P* < 0.05

TEM imaging showed that the phage particles released from *Acidithiobacillus ferrooxidans* possessed icosahedral capsids of ~100 nm in diameter, without visible tail structures (Fig. 1a). One day after phage introduction, phage abundance was 9.42 × 10^5^ VLPs mL^−1^, 150-fold higher than that in the CK group (6.26 × 10^3^ VLPs mL^−1^; Fig. 1b). Virome analysis of the purified phage inoculum identified two distinct vOTUs, AfP_1732 and AfP_5388, with contig lengths greater than 5 kb. AfP_1732 and AfP_5388 contained 12 and 11 predicted ORFs, respectively, with coding densities of 81.72% and 72.66% and GC contents of 61.32% and 31.85%, respectively (Table S1).

### Phages enhance chalcopyrite dissolution and leaching kinetics

**Fig. 2:**
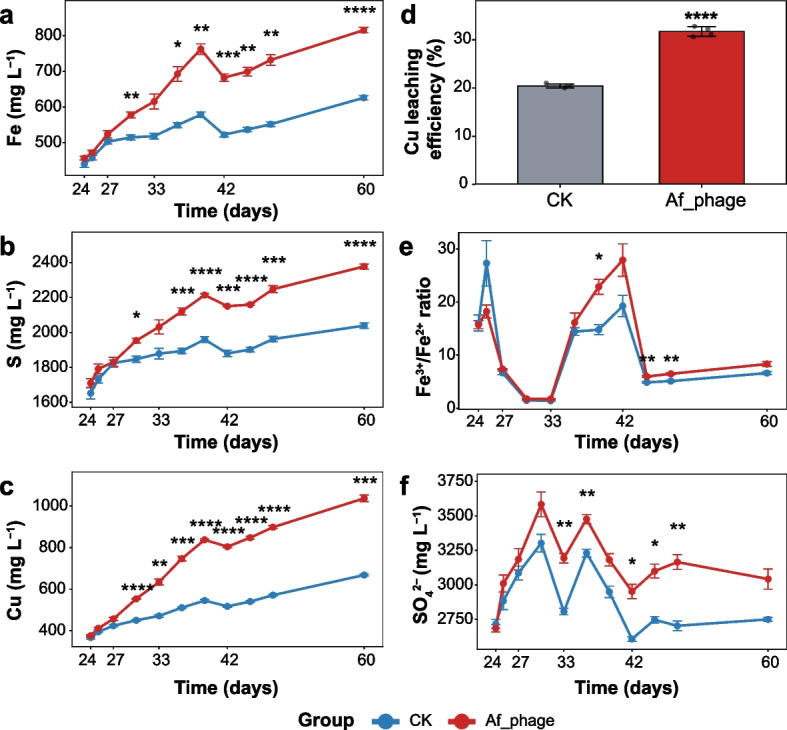
Effects of Af_phage treatment on chalcopyrite dissolution and associated physicochemical dynamics. Temporal changes in dissolved Fe (**a**), S (**b**), and Cu (**c**) concentrations, **d** copper leaching efficiency at day 60, **e** Fe^3+^/Fe^2+^ ratio, and **f** SO_4_^2−^ concentration in the CK and Af_phage groups. Data are presented as mean ± SD (n = 3). Asterisks indicate significant differences between treatments at each time point (* *P* < 0.05, ** *P* < 0.01, *** *P* < 0.001, **** *P* < 0.0001; Bonferroni-adjusted *P* values)

Phage addition markedly enhanced chalcopyrite dissolution and altered leaching kinetics. After 60 days of bioleaching, dissolved Fe, S, and Cu concentrations in the Af_phage group were higher than those in the CK, with relative increases of 30.27%, 16.60%, and 55.29%, respectively (Fig. 2a–c). The copper leaching efficiency of the Af_phage group was 31.72%, significantly higher than that of the CK group (20.43%; *P* < 0.05; Fig. 2d). By day 24, chalcopyrite dissolution became kinetically hindered, consistent with the onset of surface passivation. Phage addition rapidly restored Cu release in the Af_phage group, with dissolved Cu increasing from 366.23 mg L^−1^ to 837.34 mg L^−1^ within the subsequent 15 days (Fig. 2c). The average Cu release rate during this period in the Af_phage group reached 31.41 mg L^−1^ d^−1^, 2.65-fold higher than that in the CK group (11.85 mg L^−1^ d^−1^).

The Af_phage group also maintained a significantly higher Fe^3+^/Fe^2+^ ratio (Fig. 2e) and SO_4_^2−^ generation during the late leaching stage (Fig. 2f), indicating enhanced microbial oxidation of both iron and sulfur species. The pH remained between 1.8 and 2.0 in both treatments, with only minor differences between groups (Fig. S2).

To quantitatively evaluate the effect of phage treatment on leaching kinetics, Cu dissolution data from days 24–60 were fitted using the shrinking core model. Both the CK and Af_phage groups showed good fits to the product layer diffusion model, with R^2^ values of 0.963 and 0.973, respectively (Fig. S3). The product-layer diffusion rate constant (*k*) in the Af_phage group was 3.31 × 10^−4^ d^−1^, 3.60-fold higher than that in the CK group (9.19 × 10^−5^ d^−1^). Based on the inverse relationship between the apparent rate constant and the time required to reach a given conversion, this increase corresponds to an estimated 72.2% reduction in the leaching time (Eq. S1). Together, these results demonstrate that phages can accelerate chalcopyrite dissolution by alleviating diffusion-limited kinetics and sustaining favorable iron-sulfur transformation conditions.

### Phages alleviate chalcopyrite surface passivation

**Fig. 3:**
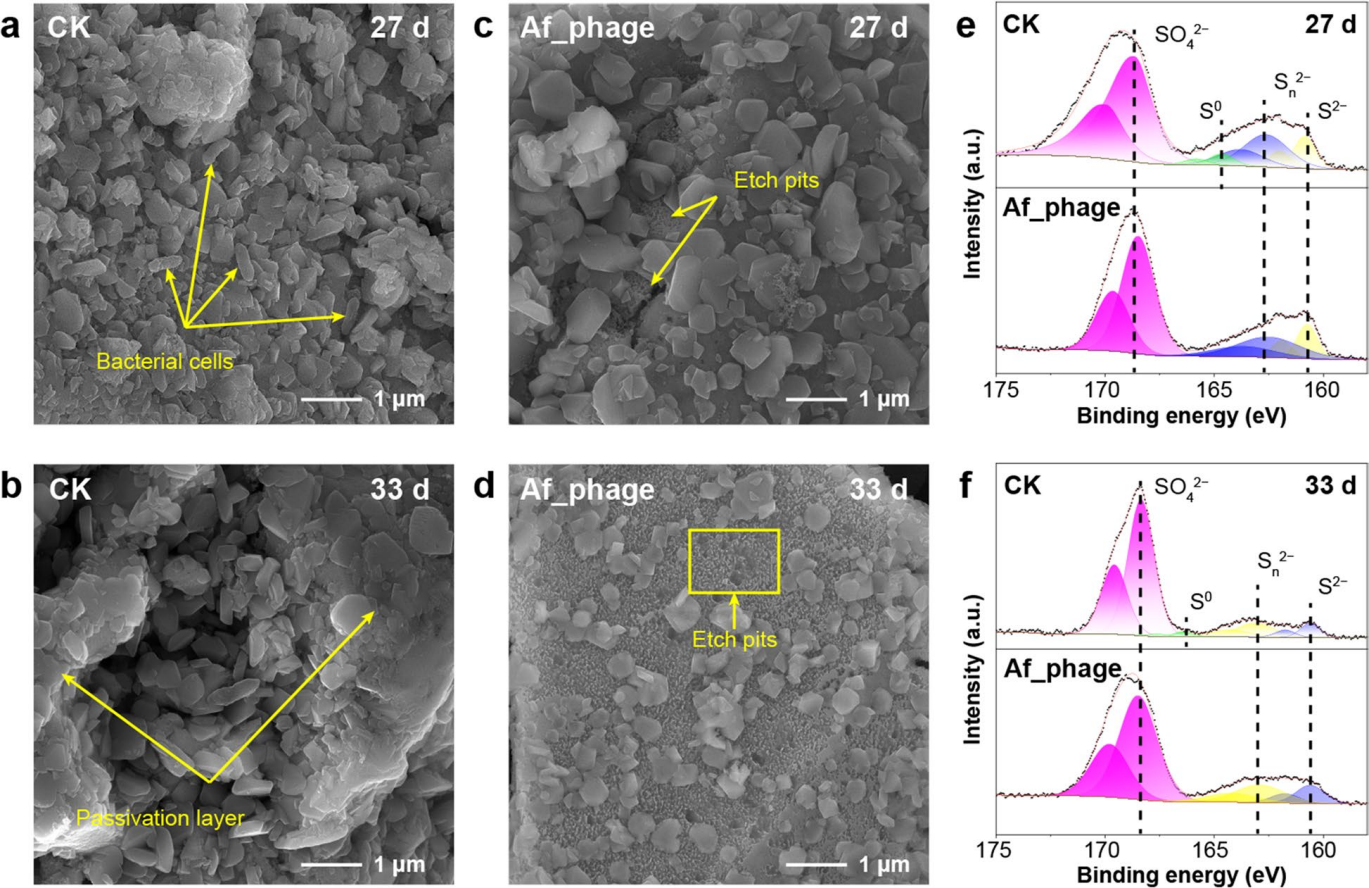
Effects of phage treatment on chalcopyrite surface passivation. **a**–**d** Representative SEM images of chalcopyrite surfaces from the CK and Af_phage groups at days 27 and 33. **e**, **f** High-resolution S 2p XPS spectra of chalcopyrite surfaces from the CK and Af_phage groups at day 27 (**e**) and day 33 (**f**). Sulfur species identified include sulfate (SO_4_^2−^), S^0^, polysulfides (S_n_^2−^), and monosulfide (S^2−^)

Af_phage treatment regulated microbe-mineral interfacial interactions and alleviated chalcopyrite surface passivation. SEM imaging revealed that the Af_phage treatment markedly reduced secondary precipitates and attached bacteria on mineral surfaces within 3–9 days after phage introduction, exposing distinct etch pits on the chalcopyrite surfaces, compared to the CK group (Fig. 3a–d).

XPS S 2p analysis showed that mineral surfaces in the Af_phage group showed less accumulation of elemental sulfur (S^0^, ~ 164.5 eV) and sulfate (SO_4_^2−^, ~ 168.5 eV) precipitates than those in the CK group (Fig. 3e and f; Table S2). In the CK group, S^0^ accounted for ~2.5% and ~4.3% of the total surface sulfur at days 27 and 33, respectively, whereas it was undetectable in the Af_phage group. FTIR analysis further revealed that mineral surfaces in the Af_phage group exhibited higher intensities of the amide I band at 1630–1642 cm^−1^ and the O–H stretching vibration at 3423 cm^−1^ than those in the CK group (Fig. S4), suggesting increased adsorption of proteinaceous substances and potentially enhanced surface hydrophilicity following phage introduction. These results indicate that phages can effectively alter the components of passivation layers on chalcopyrite surfaces.

### Phages reshape the microbial community

Viromic analysis showed that, within one day of phage introduction, the relative abundance of 17 vOTUs increased significantly (*P* < 0.05; Fig. S5), whereas the remaining vOTUs showed no significant change. By day 33, 6 of these 17 vOTUs had significantly declined, consistent with a transient rise and subsequent decline in these phage populations, as expected for active phage propagation. Notably, these enriched vOTUs were distinct from the two vOTUs in the introduced inoculum (AfP_1732 and AfP_5388), indicating that they represent resident phage populations of the consortium rather than amplification of the added phages. Among these, 12 enriched vOTUs were negatively correlated with ASVs assigned to *Acidithiobacillus* (Fig. S6), indicating selective phage suppression of the dominant autotrophic host.

**Fig. 4:**
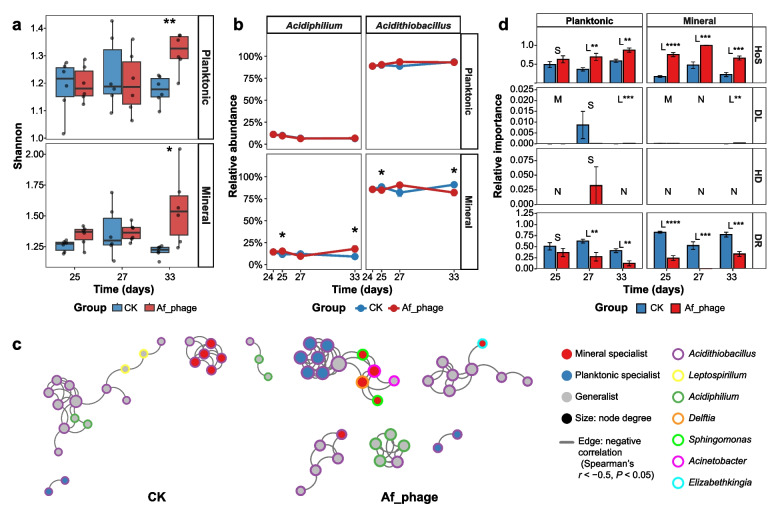
Phage-driven shifts in microbial community structure, interaction networks, and assembly processes. **a** Shannon diversity index in planktonic and mineral-associated communities. **b** Relative abundance of *Acidiphilium* and *Acidithiobacillus* in planktonic and mineral-associated phases. **c** Co-occurrence networks under CK and Af_phage treatments. Node fill color indicates ecological strategy (mineral specialists, planktonic specialists, and generalists); node border color denotes taxonomic affiliation; node size represents degree; edges denote significant negative correlations (Spearman’s *r* < − 0.5, *P* < 0.05). **d** Relative contributions of ecological assembly processes based on iCAMP: homogeneous selection (HoS), dispersal limitation (DL), homogenizing dispersal (HD), and drift (DR). Letters indicate Cohen’s d effect size (N, negligible; S, small; M, medium; L, large). Asterisks denote statistical significance (* *P* < 0.05, ** *P* < 0.01, *** *P* < 0.001, **** *P* < 0.0001). Data in **a**, **b**, and **d** are based on amplicon (16S rRNA gene) sequencing (n = 6 per group); the co-occurrence network in **c** was constructed from six matched longitudinal observations per treatment (3 biological replicate flasks ×2 time points)

Consequently, the Af_phage treatment substantially altered bacterial community structure. Within 9 days of phage addition, microbial diversity significantly increased in both the planktonic and mineral-associated communities (Fig. 4a). The relative abundance of mineral-associated *Acidithiobacillus* significantly decreased (*P* < 0.05), while that of heterotrophic *Acidiphilium* correspondingly increased (Fig. 4b). Network analysis suggested treatment-associated differences in co-occurrence patterns, including changes in connectivity and modularity (Fig. 4c and Table S3). The network was dominated by negative associations between *Acidithiobacillus* and heterotrophic taxa (*Acidiphilium*, *Delftia*, *Sphingomonas*, *Acinetobacter*), consistent with contrasting abundance patterns of *Acidithiobacillus* and heterotrophic taxa following phage introduction. Community assembly analysis indicated that, compared with the CK group, Af_phage treatment increased the relative contribution of homogeneous selection (HoS) (Fig. 4d**)**, suggesting a more deterministic ecological process shaped by phage-host interactions. Collectively, these results indicate that phage introduction altered microbial community composition, association patterns, and assembly processes during chalcopyrite bioleaching.

### Phages shift microbial functional profiles

**Fig. 5:**
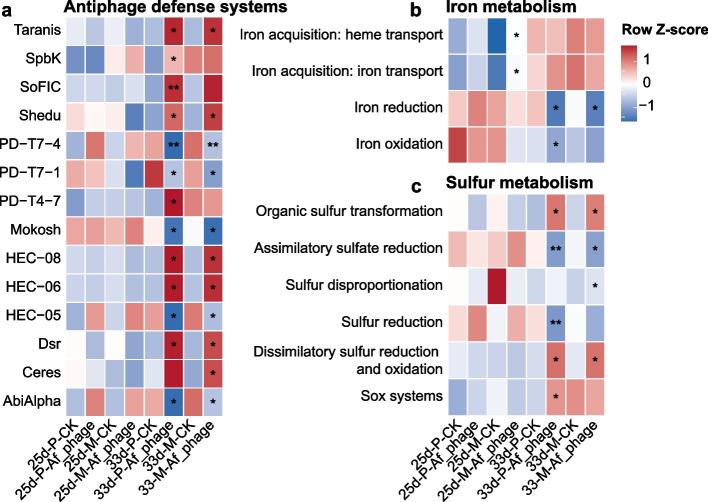
Phage-associated shifts in defense systems and metabolic functional potential during chalcopyrite bioleaching. Heatmaps showing the differential abundance of genes involved in **a** antiphage defense systems, **b** iron metabolism, and **c** sulfur metabolism across the CK and Af_phage groups in planktonic (P) and mineral-associated (M) phases on day 25 and day 33. Colors represent the row Z-score of RPKM values, indicating relative gene abundance across treatments. Asterisks denote statistical significance (* *P* < 0.05, ** *P* < 0.01). Data are based on metagenomic sequencing (n = 3)

Phage introduction shifted the functional potential of the bioleaching consortium, particularly the abundance of genes associated with antiphage defense and energy metabolism. In the Af_phage group, particularly on day 33, genes encoding multiple antiphage defense systems, including Taranis, SpbK, SoFIC, Shedu, PD-T4-7, HEC-08, HEC-06, Dsr, and Ceres, were significantly enriched (*P* < 0.05; Fig. 5a), consistent with selection for microbial populations carrying diverse antiphage defense systems.

Concurrently, phage treatment shifted the abundance profiles of genes associated with iron and sulfur metabolism. Genes associated with iron acquisition (heme transport and iron transport) were enriched in the Af_phage group at day 25, whereas genes associated with iron reduction and oxidation were significantly depleted at day 33 (Fig. 5b). In sulfur metabolism, genes involved in sulfur oxidation (Sox system) and dissimilatory sulfur metabolism were significantly enriched, while sulfur reduction was reduced (Fig. 5c). In addition, organic sulfur transformation genes were also enriched, consistent with the increased relative abundance of heterotrophic taxa observed in the Af_phage group. These results indicate that phage treatment shifted community functional potential toward sulfur transformation, consistent with enhanced sulfur turnover.

## Discussion

Our results demonstrate that the introduction of phages can alleviate surface passivation and enhance chalcopyrite bioleaching efficiency by 55.26% (from 20.43% to 31.72%) relative to the phage-free control. Phage-mediated regulation offers two key advantages for engineering applications of bioleaching. First, phages can selectively suppress or enhance specific functional populations by lysing host cells or reprogramming host metabolism, respectively (Chevallereau et al. 2022). Although phages were introduced only once in the present study, this host-specificity could, in principle, enable stage-dependent regulation of bioleaching consortia in future applications. For example, phages might be employed to promote iron-oxidizing bacteria to accelerate oxidant (Fe^3+^) regeneration during the early stage, and to enhance sulfur-oxidizing bacteria to reduce S^0^ accumulation at later stages (Feng et al. 2015). In comparison, current regulation strategies cannot simultaneously account for both the bio-oxidation activity of the microbial community and the interface reaction. For example, silver ion (Ag^+^) as a catalyst promotes Fe^3+^ attack on chalcopyrite and inhibits S^0^ formation, while Ag^+^ is toxic to bioleaching microorganisms and inhibits their metabolic activity (Nourmohamadi et al. 2022). Tween-80 enhances mineral dissolution by reducing surface tension but suppresses microbial iron oxidation activity at elevated dosages (Ghadiri et al. 2019). Second, phage-mediated regulation may exhibit self-amplifying and self-limiting behavior under conditions that support productive host infection. Phage populations can amplify as host abundance increases and become limited when host abundance falls below the lytic threshold (Payne And Jansen 2001; Suttle 2007). This density-dependent self-adaptation is particularly favorable for scale-up applications, as it could reduce the need for continuous additive supplementation. In contrast, conventional chemical or biostimulation strategies, such as activated carbon (Nakazawa et al. 1998), L-cysteine (Liu et al. 2019), and ethylene thiourea (Ren et al. 2023), require large quantities of additives to meet industrial-scale demands, often accompanied by elevated operational costs and secondary contamination concerns. These results highlight the significant potential of phages for developing novel bioleaching technologies.

Alleviation of surface passivation is a key mechanism by which phages enhance chalcopyrite bioleaching. Our results demonstrated that phage introduction suppressed S^0^ and jarosite-type precipitate accumulation and reduced bacterial cell attachment on mineral surfaces, thereby increasing interfacial ion exchange and preventing the development of passivation layers. These effects may be partly explained by phage-mediated disruption of biofilm structure. Biofilms are structured microbial communities embedded within a self-produced EPS matrix (Fulaz et al. 2019). Biofilms attached to mineral surfaces initially facilitate contact oxidation, but progressively act as a structural scaffold that traps S^0^ and jarosite precipitates, leading to the formation of passivation layers (Ma et al. 2021). Phages can actively penetrate and disrupt biofilms through diverse EPS-degrading enzymes. Phage-encoded polysaccharide depolymerases and glycoside hydrolases have been reported to degrade polysaccharide scaffolds directly, weakening the structural integrity of the EPS matrix (Hughes et al. 1998), which may help explain the biofilm disruption observed in our system. Phage infection can also facilitate cell lysis and the release of intracellular contents, leading to changes in EPS composition that influence biofilm architecture and interfacial reactivity. Collectively, these effects contribute to a reduction in polysaccharide-dominated structures and a relative increase in proteinaceous components within EPS, thereby influencing interfacial reactivity.

The introduction of phages may also promote mineralization of secondary precipitates and reduce their attachment on mineral surfaces. Evidence suggests that phage capsids possess reactive surface sites that bind metal ions (e.g., Fe^2+^/Fe^3+^) and facilitate the nucleation of iron minerals (Daughney et al. 2004). In sulfide systems, phages serving as ion-adsorbing templates improve the formation of framboidal pyrite (Działak et al. 2022). Phage presence has further been shown to alter mineral particle size distributions and composition during iron-bearing mineral precipitation (Działak et al. 2024). These findings suggest that phages can modulate mineralization pathways and may reduce the formation of iron-containing aggregates. Such mechanisms could partly explain why phage introduction reduced the attachment of passivation layers on mineral surfaces in bioleaching systems. Consistent with reduced precipitate deposition, the higher dissolved Fe, S, and SO_4_^2−^ in the Af_phage group are best understood as a consequence of this depassivation. The disruption of the biofilm/precipitate barrier released surface-bound Fe and S into solution and restored interfacial oxidation, thereby increasing the dissolved ion concentrations. The elevated Fe^3+^/Fe^2+^ ratio in the Af_phage group should therefore be interpreted as a consequence of restored iron-oxidizing activity after disruption of the biofilm/precipitate barrier, rather than as evidence for enhanced electrochemical passivation of the mineral surface. Although our results demonstrate that phages can suppress the accumulation of passivation substances on mineral surfaces, the regulatory mechanisms governing phage interactions with biofilms and passivation layers at the mineral interface warrant further investigation.

Phage-driven restructuring of bacterial communities also contributes to enhanced chalcopyrite bioleaching. Phage introduction increased microbial diversity and altered community assembly during bioleaching. During bioleaching, microbial diversity typically declines over time as dominant populations (i.e., *Acidithiobacillus*) monopolize key nutrients, such as nitrogen, restricting the coexistence of other species. Phage pressure on dominant populations can release cellular resources and alter competitive relationships, potentially supporting the coexistence of other taxa (Li et al. 2024). Consistently, our results showed that phage introduction significantly decreased the relative abundance of *Acidithiobacillus* on mineral surfaces (*P* < 0.05), while increasing that of *Acidiphilium*. The two taxa were clustered within the same co-occurrence module. It has been reported that co-culture of *Acidithiobacillus ferrooxidans* and *Acidiphilium acidophilum* greatly promotes bioleaching performance, because *Acidiphilium* can heterotrophically utilize organic substrates and alleviate metabolic inhibition of autotrophic *Acidithiobacillus* (Liu et al. 2011). Similarly, *Acidiphilium cryptum* can enhance the dissolution of manganese and rock phosphate with *Acidithiobacillus*, likely through metabolic regulation of pH and redox conditions (González et al. 2018). Moreover, phage introduction altered the ecological associations between *Acidithiobacillus* and heterotrophic species on mineral surfaces, such as *Sphingomonas* and *Acinetobacter*. These heterotrophic species may play critical functional roles in mitigating passivation at mineral interfaces. For example, *Sphingomonas* possesses the metabolic potential to degrade organometallic compounds (Asaf et al. 2020), which may help destabilize EPS-metal complexes within the passivation layer (d’Abzac et al. 2010; Ma et al. 2021). *Acinetobacter* spp. can promote biofilm formation through quorum sensing molecules under metal-rich conditions (Sarkar And Chakraborty 2008), potentially facilitating reattachment of leaching bacteria and subsequent mineral dissolution. Overall, these findings indicate that phages reshape surface microbial communities and shift the relative abundance patterns and ecological associations of bioleaching bacteria and heterotrophic taxa, ultimately enhancing bioleaching performance.

Phage treatment shifted the community functional potential toward sulfur transformation, consistent with enhanced sulfur turnover and reduced S^0^ deposition. We found that phage introduction significantly enriched sulfur oxidation genes in the microbial community, consistent with reduced S^0^ accumulation on mineral surfaces and improved bioleaching efficiency. Recent studies have reported that phages encode diverse genes for sulfur oxidation across various environments, including *dsrA*, *soxCD*, and *soxYZ* (Kieft et al. 2021; Li et al. 2021). These genes are capable of accelerating rate-limiting steps in the oxidation of S^0^ and thiosulfate. Phages can also promote the conversion of inorganic sulfur to organic forms by participating in sulfur assimilation through *cysH* and *mec* genes (Hesketh-Best et al. 2023). Together, these observations support a phage-associated shift toward sulfur turnover at the mineral surface. We note, however, that sulfur-metabolizing auxiliary metabolic genes were not detected in the phages of this study. The enhanced sulfur oxidation is therefore attributed to phage-mediated restructuring of the host community rather than to phage-encoded sulfur genes. Further work is needed to resolve how phage-host interactions regulate sulfur metabolism in bioleaching consortia.

It should be noted that the present study was conducted at flask scale and at a low pulp density (1% w/v), a condition deliberately chosen to enable clear, well-controlled observation of the interfacial passivation dynamics that are the focus of this work. Chalcopyrite is highly refractory to leaching owing to surface passivation, and copper recovery from chalcopyrite typically remains limited under mesophilic conditions. The copper bioleaching efficiency achieved here (31.72% over 60 days, a relative increase of 55.26% over CK) represents a meaningful improvement for such a refractory mineral. Applying phage-mediated regulation to industrial heap or stirred-tank bioleaching, which is characterized by high pulp densities, strong acidity, elevated ionic strength and metal concentrations, and heterogeneous mineral surfaces, will require further investigation of phage persistence, infectivity, and host encounter under such conditions.

## Conclusion

To our knowledge, this study provides the first evidence that phage introduction can alleviate surface passivation and enhance chalcopyrite bioleaching. Phages released from *Acidithiobacillus ferrooxidans* increased copper leaching efficiency by 55.26% relative to phage-free controls (31.72% vs. 20.43%). Phage treatment reduced biofilm-associated surface coverage and the accumulation of S^0^ and jarosite-type passivation products, while increasing the apparent product-layer diffusion rate constant. It also reshaped the microbial community and enriched genes associated with sulfur oxidation and transformation, consistent with enhanced S^0^ turnover. Together, these findings reveal a coupled interfacial-microbial mechanism by which phage introduction alleviates passivation, promotes sulfur turnover, and enhances chalcopyrite bioleaching.

## Supplementary Information

Supplementary Material 1.

## Data Availability

The raw sequencing data generated in this study, including 16S rRNA gene amplicon, shotgun metagenomic, and viromic sequencing reads, have been deposited in the NCBI Sequence Read Archive (SRA) under BioProject accession PRJNA1474737.
