# Supplementary material for: Phages released from Acidithiobacillus ferrooxidans enhance chalcopyrite bioleaching by alleviating passivation and promoting sulfur turnover
Source: Adv Biotechnol (Singap). 2026 Jul 27;4(3):29. doi: 10.1007/s44307-026-00122-x (PMC13407799; doi:10.1007/s44307-026-00122-x)
Supplement: Supplementary file 1 — Supplementary Material 1. [file 44307_2026_122_MOESM1_ESM.docx]

**Supporting Information**

Phages released from *Acidithiobacillus ferrooxidans* enhance chalcopyrite bioleaching by alleviating passivation and promoting sulfur turnover

Zhaoyue Yang^a^, Zhenghua Liu^a^, Delong Meng^a^, Zhengdong Yang^b^, Kuojun Hu^a^, Zhuzhong Yin^a^, Ling Xia^c^, Ibrahim Ahmed Ibrahim^d^, Xiangdong Xiao^e^, Xueduan Liu^a^, Huaqun Yin^a*^

^a^School of Minerals Processing and Bioengineering, Central South University, Changsha, 410083, China

^b^School of Architecture and Civil Engineering, Chengdu University, Chengdu, 610106, China

^c^Hubei Key Laboratory of Mineral Resources Processing and Environment, School of Resources and Environmental Engineering, Wuhan University of Technology, Wuhan, 430070, China

^d^Central Metallurgical Research and Development Institute, Cairo, 11421, Egypt

^e^Hunan Yama Biotechnology Co., Ltd., Changsha, 410000, China

**^*^Corresponding author:**

Huaqun Yin

E-mail: [yinhuaqun_cs@sina.com](mailto:yinhuaqun_cs@sina.com)

School of Minerals Processing and Bioengineering, Central South University, Changsha, 410083, China

**Supplementary** **figures**

**
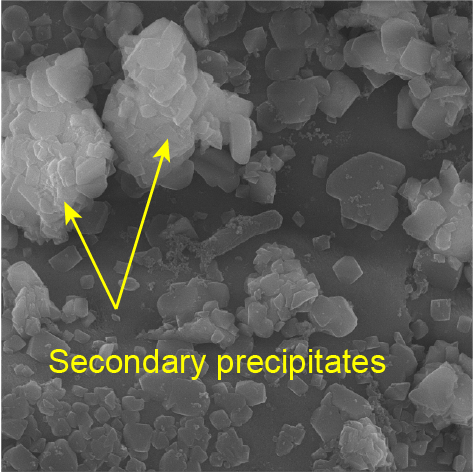
**

**Fig. S1** Scanning electron microscopy (SEM) image of chalcopyrite surfaces at day 24 of bioleaching. Dense deposits of secondary precipitates and attached microbial cells were observed on mineral surfaces, indicating the onset of surface passivation. This time point was used to define the timing of phage addition in the Af_phage group


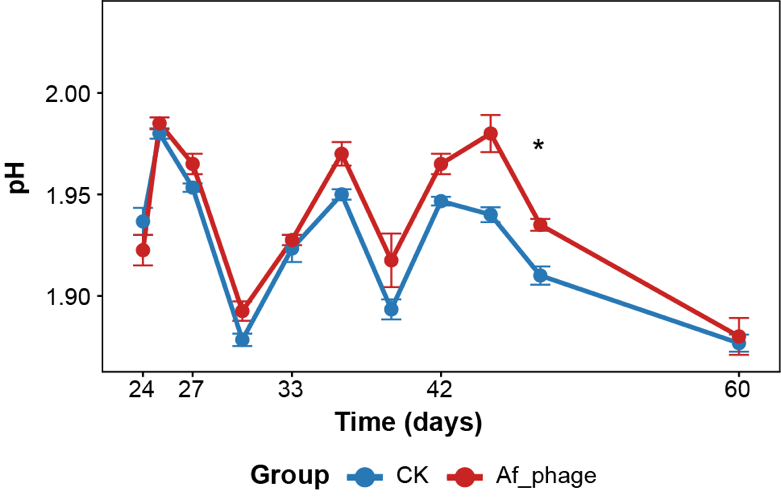


**Fig. S2** Temporal changes in pH in the CK and Af_phage groups during 60 days of chalcopyrite bioleaching. Data are presented as mean ± standard deviation (n = 3). An asterisk indicates *P* < 0.05


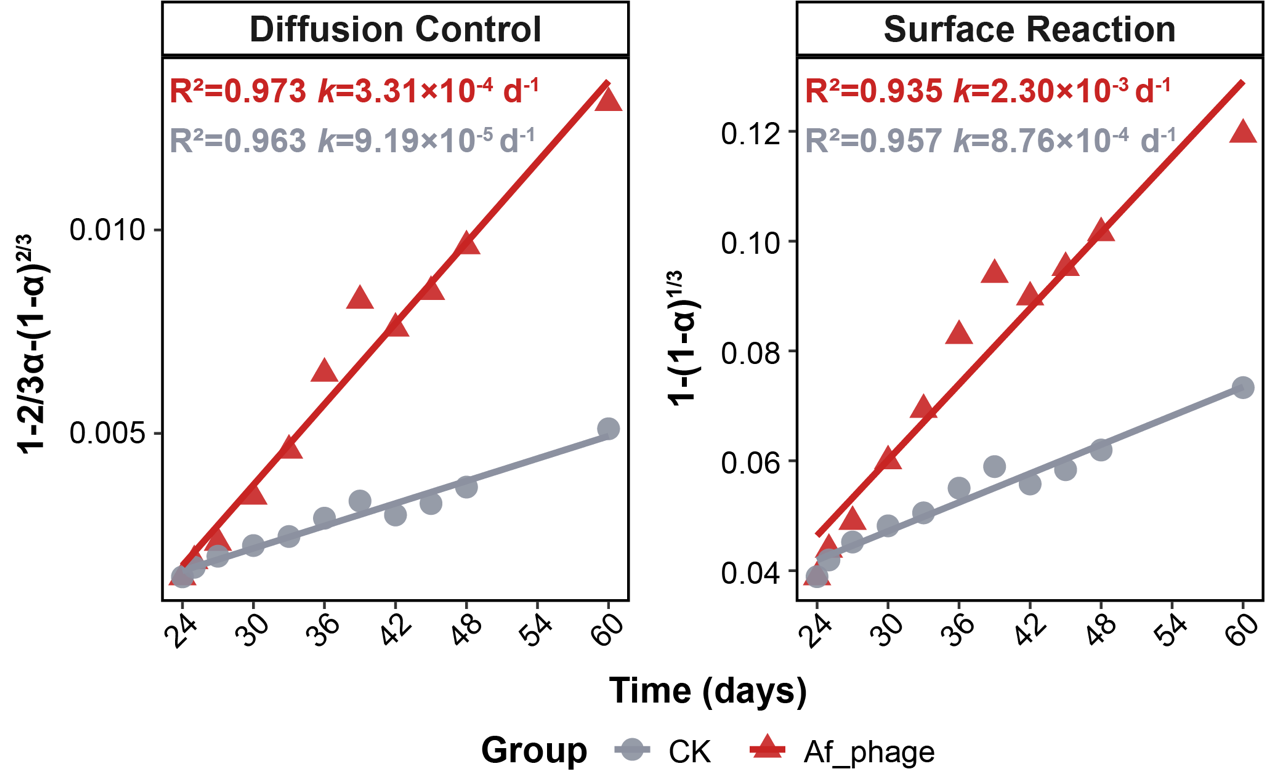


**Fig. S3** Kinetic analysis of chalcopyrite bioleaching using the shrinking core model (SCM). Cu dissolution data from days 24 to 60 were fitted to the product layer diffusion control model: ${1-\frac{2}{3}\alpha-\left( 1-\alpha\right)}^{2/3}=kt$, and the surface reaction control model: ${1-\left( 1-\alpha\right)}^{1/3}=kt$, where α represents the Cu extraction efficiency. The apparent rate constants (*k*, d^−1^) and coefficients of determination (R^2^) for the CK and Af_phage groups are indicated in each panel


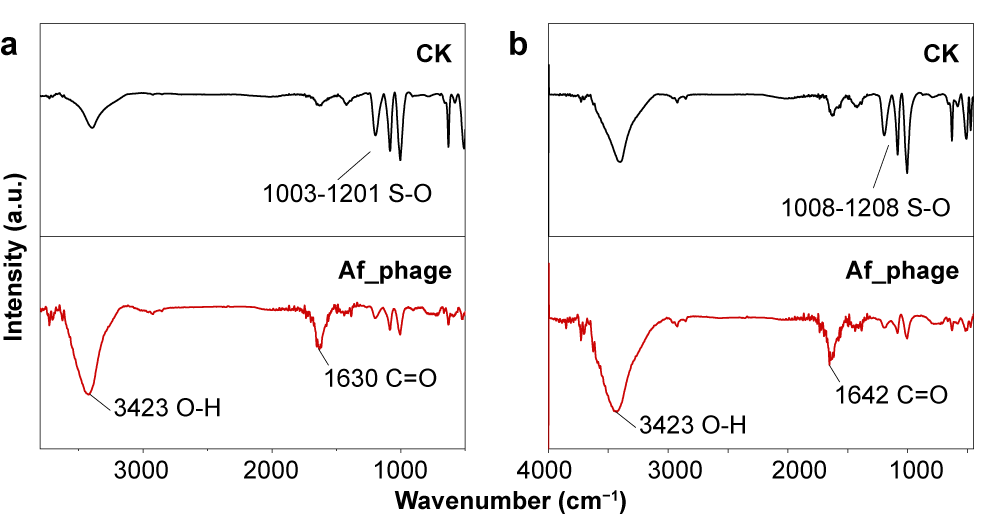


**Fig. S4** Fourier transform infrared spectroscopy (FTIR) analysis of functional groups on chalcopyrite surfaces after Af_phage treatment. FTIR spectra from the CK and Af_phage groups at day 27 (a) and day 33 (b). Characteristic bands corresponding to O-H stretching, C=O stretching, and S-O vibrations are labeled


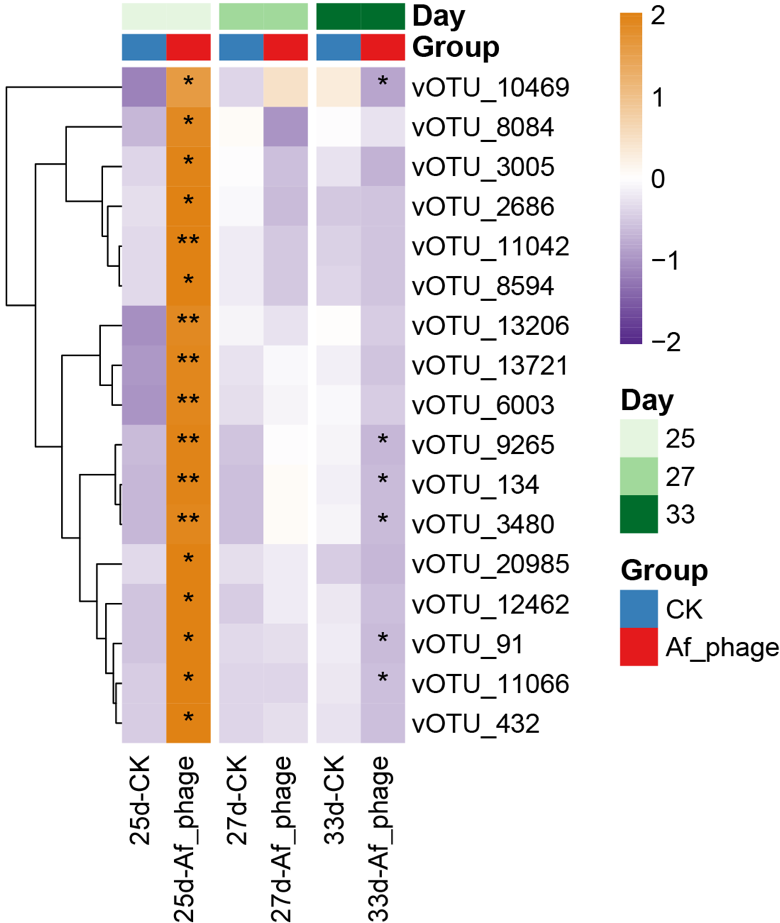


**Fig. S5** Phage-induced enrichment of viral operational taxonomic units (vOTUs) in the bioleaching system. Heatmap showing the relative abundance of vOTUs in the CK and Af_phage groups at different time points during bioleaching. Significant enrichment in the Af_phage group compared with the CK group is indicated by asterisks (* *P* < 0.05, ** *P* < 0.01). A total of 17 vOTUs were significantly enriched following phage introduction. By day 33, 6 of these 17 vOTUs had significantly declined


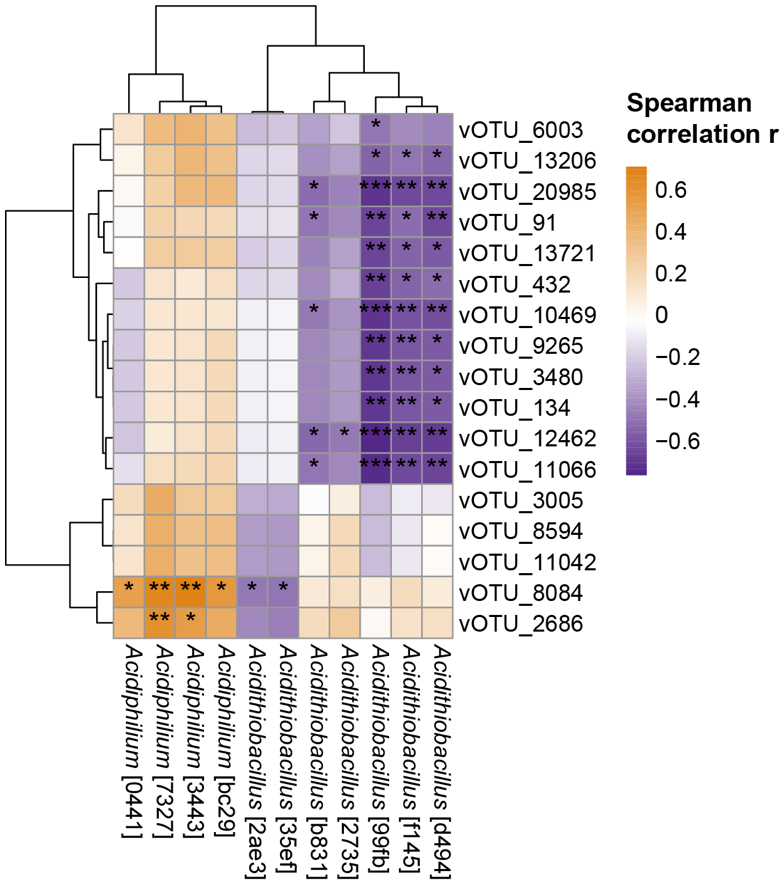


**Fig. S6** Correlations between 17 enriched vOTUs and bacterial ASVs during chalcopyrite bioleaching. Statistically significant correlations are marked (* *P* < 0.05, ** *P* < 0.01). Twelve vOTUs exhibited significant negative correlations with *Acidithiobacillus* ASVs

**Supplementary tables**

**Table S1** Genomic features of bacteriophage vOTUs released from *Acidithiobacillus ferrooxidans* identified in this study

| **vOTU**  **ID** | **Length**  **(bp)** | **GC content**  **(%)** | **ORF count** | **Coding density**  **(%)** | **CheckV**  **quality** | **Completeness**  **(%)** |
| --- | --- | --- | --- | --- | --- | --- |
| AfP_1732 | 6854 | 61.32 | 12 | 81.72 | Low-quality | 8.98 |
| AfP_5388 | 6565 | 31.85 | 11 | 72.66 | Low-quality | 15.10 |

**Table S2 Surface sulfur speciation from XPS S 2p deconvolution**

| **Day** | **Group** | **Monosulfide**  **(~160.7 eV)** | **Polysulfide**  **(~162.7 eV)** | **Elemental S^0^**  **(~164.5 eV)** | **Sulfate**  **(~168.5 eV)** |
| --- | --- | --- | --- | --- | --- |
| 27 | CK | 9.1 | 18.9 | 2.5 | 69.6 |
| 27 | Af_phage | 11.7 | 21.5 | n.d. | 66.8 |
| 33 | CK | 4.3 | 8.3 | 4.3 | 83.1 |
| 33 | Af_phage | 9.6 | 16.3 | n.d. | 74.0 |

Note: Values are percentages of total surface sulfur; n.d., not detected.

**Table S3** Topological properties of bacterial co-occurrence networks in the CK and Af_phage groups

| **Group** | **Nodes** | **Edges** | **Average degree** | **Modularity** |
| --- | --- | --- | --- | --- |
| CK | 25 | 38 | 3.04 | 0.63 |
| Af_phage | 32 | 60 | 3.75 | 0.69 |

**Supplementary equation**

In the product-layer diffusion model, the conversion function is related to time by:

${1-\frac{2}{3}\alpha-\left( 1-\alpha\right)}^{2/3}=kt$ (S1)

For a given target conversion (*α*), the left-hand side is a constant, so the time required to reach that conversion is inversely proportional to the apparent rate constant *k*. The ratio of the times required by the CK and Af_phage groups to reach the same conversion is therefore:

$\frac{t_{Af\_phage}}{t_{CK}}=\frac{k_{CK}}{k_{Af\_phage}}=\frac{9.19\times{10}^{-5}}{3.31\times{10}^{-4}}=0.278$

corresponding to a reduction in leaching time of 1 − 0.278 = 72.2%.
